# Precise Planar-Twisted Molecular Engineering to Construct Semiconducting Polymers with Balanced Absorption and Quantum Yield for Efficient Phototheranostics

**DOI:** 10.34133/research.0194

**Published:** 2023-07-26

**Authors:** Xiang Su, Zhirong Bao, Wei Xie, Deliang Wang, Ting Han, Dong Wang, Ben Zhong Tang

**Affiliations:** ^1^ Center for AIE Research, Shenzhen Key Laboratory of Polymer Science and Technology, Guangdong Research Center for Interfacial Engineering of Functional Materials, College of Materials Science and Engineering, Shenzhen University, Shenzhen 518060, China.; ^2^ School of Biomedical and Pharmaceutical Sciences, Guangdong University of Technology, Guangzhou 510006, China.; ^3^ Department of Radiation and Medical Oncology, Hubei Key Laboratory of Tumor Biological Behaviors, Hubei Cancer Clinical Study Center, Zhongnan Hospital of Wuhan University, Wuhan 430071, China.; ^4^ Department of Materials Chemistry, Huzhou University, Huzhou 313000, China.; ^5^ School of Science and Engineering, Shenzhen Institute of Aggregate Science and Technology, The Chinese University of Hong Kong, Shenzhen, Guangdong 518172, China.

## Abstract

Semiconducting polymers (SPs) have shown great feasibility as candidates for near-infrared-II (NIR-II) fluorescence imaging-navigated photothermal therapy due to their strong light-harvesting ability and flexible tunability. However, the fluorescence signal of traditional SPs tends to quench in their aggregate states owing to the strong π–π stacking, which can lead to the radiative decay pathway shutting down. To address this issue, aggregation-induced emission effect has been used as a rational tactic to boost the aggregate-state fluorescence of NIR-II emitters. In this contribution, we developed a precise molecular engineering tactic based on the block copolymerizations that integrate planar and twisted segments into one conjugated polymer backbone, providing great flexibility in tuning the photophysical properties and photothermal conversion capacity of SPs. Two monomers featured with twisted and planar architectures, respectively, were tactfully incorporated via a ternary copolymerization approach to produce a series of new SPs. The optimal copolymer (SP2) synchronously shows desirable absorption ability and good NIR-II quantum yield on the premise of maintaining typical aggregation-induced emission characteristics, resulting in balanced NIR-II fluorescence brightness and photothermal property. Water-dispersible nanoparticles fabricated from the optimal SP2 show efficient photothermal therapeutic effects both in vitro and in vivo. The in vivo investigation reveals the distinguished NIR-II fluorescence imaging performance of SP2 nanoparticles and their photothermal ablation toward tumor with prominent tumor accumulation ability and excellent biocompatibility.

## Introduction

Phototheranostics, harnessing photon energy to output fluorescence, acoustic signals, reactive oxygen species and/or hyperthermia, has emerged as an enticing solution to cancer by dint of the integrated superiorities of light prompted noninvasiveness and real-time monitoring as well as in situ manipulative treatment [1–5]. As an imaging tool conducted in near-infrared-II (NIR-II) (1,000 to 1,700 nm) window, NIR-II fluorescence imaging (NIR-II FLI) serves as a burgeoning and representative diagnostic technique, rendering copious biological feedback from deeper lesion locations with higher signal-to-noise ratio relative to NIR-I (700 to 900 nm) FLI. The good performance of NIR-II FLI mainly arises from the deep penetration depth of NIR-II light and slight absorption together with the diminished photoscattering and imperceptible autofluorescence of biological tissues in NIR-II window [6–9]. As an effective photo-driven therapeutic modality, photothermal therapy (PTT) takes advantage of the heat produced from photothermal agents for tumor ablation, in which photothermal agents convert the absorbed photon power into hyperthermia on specific lesion sections upon light excitation. Compared to the conventional therapeutic modalities such as surgical excision, chemotherapy, and radiotherapy, the PTT technique enjoys the merits of light controllability, noninvasiveness, and high effectiveness [10–13]. Attracted by the advantages of NIR-II FLI and PTT, multifunctional nanoplatforms with integrative NIR-II FLI capacity and PTT property have gained extensive attention as a promising tactic for precise tumor theranostics with minimal harm to human health [14–17].

To meet the requirements of NIR-II FLI-assisted PTT theranostics, it is extremely urgent to develop exogenous contrast agents with efficient NIR-II emission and high photothermal effectiveness through precise molecular engineering. Up to now, a variety of inorganic nanomaterials, such as carbon nanotubes [18], quantum dots [19–20], and rare earth nanoparticles (NPs) [21,22], have been widely explored for NIR-II FLI, but their long-term biotoxicity and minimal biodegradability could substantially hinder their practical applications [23]. Small molecular organic dyes exhibit superiority in the aspect of biocompatibility, but they often suffer from unsatisfactory photostability and limited accumulation capacity [24,25]. In contrast, semiconducting polymers (SPs) with large π-conjugated architectures exhibit reliable biosafety, strong light-harvesting ability, durable photostability, excellent photothermal conversion ability, etc. They have manifested great talents in the fields of FLI, photoacoustic imaging, and imaging-guided PTT and/or photodynamic therapy [26–33]. In addition, the flexible tunability of the polymer skeleton endows SPs with versatile optical properties and therapeutic properties [34]. Hence, SPs are promising candidates for NIR-II FLI-guided PTT toward antitumor applications. However, currently, the structural diversity SP-based NIR-II emitters are still very limited, and their fluorescence performance still needs to be further improved.

On the basis of the Jablonski diagram, photoexcited dyes can dissipate their excited state energy through the radiative pathway to benefit fluorescence or through nonradiative channel to boost photothermal conversion. However, the radiative and nonradiative attenuations are 2 competitive channels involved in FLI and photothermal properties. Extending the conjugation length of organic probes or using highly conjugated SPs has been used as a feasible tactic to realize the red shift of emission wavelength. Nevertheless, the fluorescence of such materials tends to quench in the aggregate state with the presence of strong intermolecular π–π stacking interactions, which could hinder the occurrence of the radiative decay pathway [35]. To address this challenge, recently, researchers have highlighted several reliable approaches to promoting the NIR-II fluorescence brightness of SPs by adjusting the structures of polymer backbones, such as the introduction of the rigid 3-dimensional moieties and the bulky π-bridge onto the polymer backbone to prevent the aggregation-quenched fluorescence phenomenon via the effective steric hindrance [36], the incorporation of a weak electron-donating unit into the conjugated polymeric skeleton with strong electron donor–acceptor (D–A) alternated structure [37], “electron acceptor density adjustment” strategy [38], double-acceptor integration into the conjugated backbone [39], and the tactic of increasing the number of the polymer repeating unit [40]. In addition, some approaches focused on reducing intermolecular interactions by enhancing steric hindrance through side chain engineering have also been proposed to boost the NIR-II fluorescence brightness, including the adjustment of the substituted locations, configuration, and size of the SP side chains as well as the fluorination chemistry [41–43]. Besides the abovementioned strategies, another promising tactic aimed at exploiting aggregation-induced emission luminogens (AIEgens) to block the fluorescence quenching of traditional NIR-II emitters in the aggregated states [44–45]. According to the well-accepted working mechanism of AIE, the design philosophy of AIEgens mainly relies on the backbone distortion and the introduction of twisted molecular motors, which can contribute to the remarkable restriction of the intermolecular π–π interactions for high fluorescence quantum yield (QY) [46–52]. However, the inherently twisted structure could remarkably decrease the conjugation extent of SPs, resulting in inferior absorption coefficient and photothermal properties. With the aim of simultaneously achieving strong NIR-II fluorescence of the aggregation states and satisfactory photothermal behavior, one possible approach could be prolonging conjugation extent to increase the absorption extinction coefficient on the basis of the guaranteed AIE feature. Previously, our group has tried to combine the planar and twisted units into one polymer skeleton through a 2-component D–A polymerization strategy to improve the light-harvesting capacity and NIR-II QY simultaneously [53]. However, this integration strategy relied on the presynthesis of Janus monomers with the planar and twisted segments in different sides via the stepwise sequential introduction of different functional segments. When the slight variation of any segment is required to tune the optical and PTT behaviors of SPs, tedious and repeated synthetic protocols have to be conducted from scratch to produce the expected polymers. Thus, such a Janus-monomer-based engineering strategy is sophisticated, time-consuming, and relatively difficult to be generalized. In addition, NIR-II FLI and photothermal conversion behaviors were attributed to various factors, including absorption wavelength, extinction coefficient, luminescent ability, and stacking manners of nanoaggregate states. It has remained to be challenging for the balance between fluorescence output and photothermal behavior based on one specific component. Thus, the development of a convenient and versatile molecular engineering strategy that can precisely modulate and optimize molecular absorption band, light-harvesting capability, and photothermal performance of SPs will be of great meaning to motivate the advancement of highly effective NIR-II emitters with satisfying PTT effect.

**Fig. 1.**
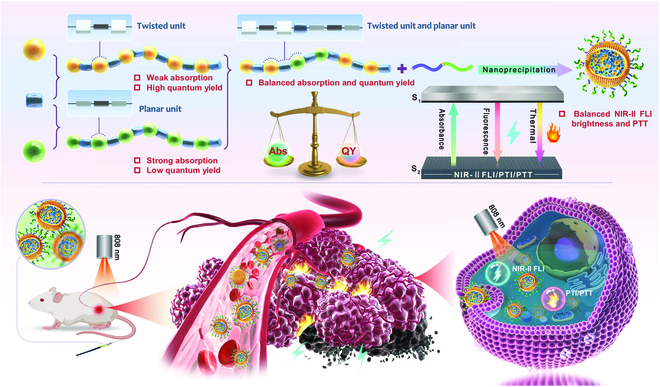
Schematic illustration of the polymer design principle, NP fabrication, and the potential application of the optimized block copolymer with balanced absorption and NIR-II QY in imaging-guided phototheranostics.

In this contribution, we developed a precise molecular engineering tactic based on block copolymerization to construct AIE SPs with strong NIR-II fluorescence and satisfactory photothermal behavior in vivo. As displayed in Fig. 1, we tactfully incorporated twisted monomer T1-BBTD-T1 with ortho-positioned alkyl units, as well as coplanar monomer T2-BBTD-T2 with meta-positioned alkyl units, via a ternary copolymerization approach to producing SPs. The photophysical properties and photothermal conversion capacity can be flexibly tuned by precise regulation of the feed ratio of the 2 monomers without the requirement of tedious presynthesis of complex Janus monomers. The optimized copolymer named SP2 with a molar feed ratio of 0.3:0.7 for T1-BBTD-T1 and T2-BBTD-T2 monomers was attained. This copolymer exhibited typical AIE characteristics and possessed high absorbance at 808 nm and satisfactory NIR-II QY, resulting in high-quality NIR-II FLI in vivo. The ceiling of output signals of a fluorescent emitter was determined by the absorption ability, and the high absorption capacity can provide elevated radiative attenuation for FLI and reinforced nonradiative decay for photothermal ablation. Thus, SP2 possessed more prominent PTT effectiveness relative to the entirely twisted polymer SP1 and exhibited excellent performance in 808-nm laser-excited NIR-II FLI-navigated photothermal elimination of tumors in the 4T1-tumor-bearing BALB/c nude mice model. The remarkable biocompatibility of SP2 NPs, verified by the histological and immunohistochemical analysis, endows them with good potential for future clinical applications.

## Results and Discussion

### Polymer design principle and theoretical calculation

Aiming at constructing NIR-II fluorophores, we design the SPs with typical strong electron D–A type composition, which will result in the highly separated electron clouds between the highest occupied molecular orbital (HOMO) and the lowest unoccupied molecular orbital (LUMO), thus leading to a narrow band gap and boosting the red shift of wavelength. Compared with the widely used triphenylamine donor core, phenothiazine (PTZ) containing the sp^3^-hybridized sulfur and nitrogen atoms has superior electron-donating ability and exhibits an excellent nonplanar boat shape configuration. Hence, the introduction of PTZ unit in polymer structures would be favorable for hindering π–π stacking aggregation and excimer formation, thereby contributing to the enhancement of aggregate-state fluorescence for the resulting conjugated polymers [54–55]. Accordingly, we used a PTZ derivative as the donor unit in the polymer backbone to elevate the NIR-II fluorescence efficiency. Meanwhile, the benzo[1,2-c:4,5-c′]bis([1,2,5]thiadiazole) (BBTD) with strong electron-withdrawing capability was used as the electron-accepting segment to pull down the LUMO level [56]. Besides, thiophene was added adjacent to the BBTD core as a donor/π-conjugated unit to further facilitate the intramolecular charge transfer process and reduce the energy bandgap of the repeating units. Moreover, to improve the solubility, regulate the configuration, and finely tune the photophysical properties of the SPs, we tactfully manipulated the location of alkyl chain on the thiophene moiety. For instance, the alkyl groups located at the meta-position of thiophene could lead to a coplanar T-BBTD-T module due to the inherent spatial distance between the alkyl side chains with the BBTD unit, whereas ortho-located alkyl chains may contribute large steric hindrance to the BBTD core to acquire a twisted backbone structure.

**Fig. 2.**
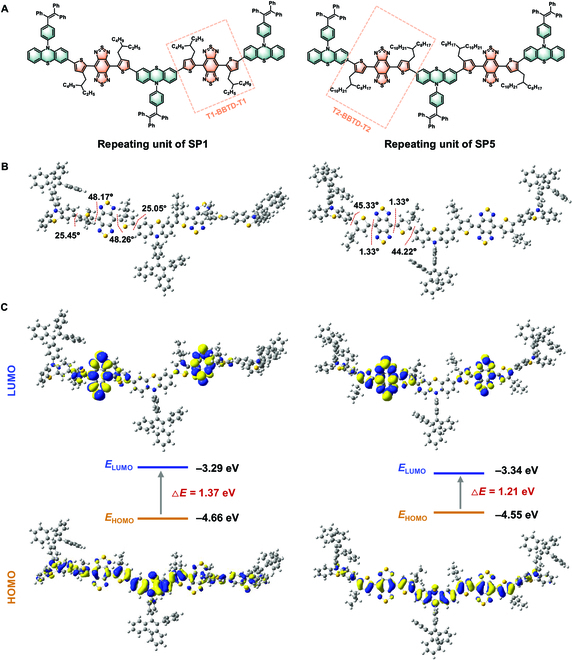
(A) Chemical constructions, (B) optimal ground-state (S_0_) geometries, and (C) diagram of the LUMO and HOMO energy levels of simplified SP1 and SP5 structures through the B3LYP/6-31G (d,p) procedure.

To verify the viability of the molecular design, we conducted the density functional theory calculations through the B3LYP/6-31G (d,p) procedure in Gaussian 09 software to estimate the electronic distribution and geometries of designed 2 D–A–D–A–D repeating units (simplified structures of SP1 and SP5) based on the PTZ moiety and T1-BBTD-T1 and T2-BBTD-T2 moieties, respectively (Fig. 2A). The S_0_ geometries in Fig. 2B disclosed the dihedral angles between the BBTD core and thiophene spacer in SP1 repeating unit were 48.17° and 48.26°, respectively, indicating a distorted backbone that could block the intermolecular interactions and implement a promoted NIR-II fluorescence QY in the aggregation. In contrast, the T2-BBTD-T2 module in SP5 repeating unit exhibited a nearly coplanar structure from the feedback of the small dihedral angle of 1.33°. In addition, the dihedral angles between the thiophene spacer and the PTZ moiety of SP1 and SP5 were measured to be about 25° and 45°, respectively, owing to the different spatial distance between the alkyl substituent and the PTZ donor. As shown in Fig. 2C, the electron distribution of the LUMO in the simplified structures of SP1 and SP5 was dominantly localized on the BBTD unit. While the HOMO was delocalized across the whole conjugated skeleton, suggesting a strong D–A effect and intramolecular charge transfer derived from the donor/π-conjugated thiophene and PTZ units to the electron-accepting BBTD core. In particular, the smaller energy gap of SP5 repeating unit also indicated a red-shifted absorption because of the incorporation of coplanar T-BBTD-T architecture. Encouraged by the distinct electron distribution and geometries of the simulated SP1 and SP5 repeating units, we then tried to precisely regulate the molar ratios of the 2 kinds of T-BBTD-T units and the resulting intramolecular charge transfer effect in polymer backbones with the aim of experimentally optimizing the NIR-II fluorescence QY, absorption wavelength, and extinction coefficient of NIR-II SPs.

### Synthesis and photophysical properties of SPs

**Fig. 3.**
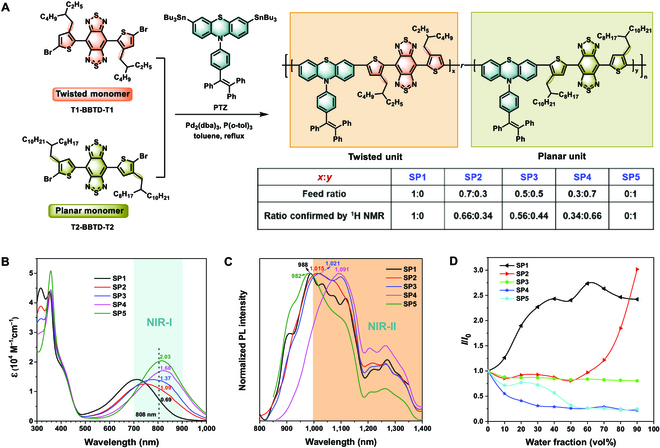
(A) Synthesis and chemical structures of polymers SP1 to SP5. (B) Absorption spectra and (C) normalized PL spectra of the THF solutions of SP1 to SP5 (10 μM). (D) Plots of the relative PL intensity (*I*/*I*_0_) of SP1 at 988 nm, SP2 at 1,015 nm, SP3 at 1,099 nm, SP4 at 1,091 nm, and SP5 at 982 nm versus H_2_O fraction in THF/H_2_O mixtures.

Monomer PTZ was synthesized according to the procedures shown in Fig. S1, and the other 2 BBTD-containing monomers were commercially available. As illustrated in Fig. 3A, the Stille polycondensation between monomers PTZ and T1-BBTD-T1 (twisted monomer) or T2-BBTD-T2 (planar monomer) yielded the copolymers SP1 and SP5, respectively, while the random copolymerizations of monomers PTZ, T1-BBTD-T1, and T2-BBTD-T2 led to the ternary block copolymers SP2 to SP4. By precisely varying the feed molar ratios of these 3 monomers, the photophysical properties of the obtained copolymers were anticipated to be tuned accordingly. The chemical structures of SP1 to SP5 were defined by gel permeation chromatography investigation and ^1^H nuclear magnetic resonance (NMR) spectroscopy. The gel permeation chromatography results showed that the weight-average molecular weight (*M*_w_) of the obtained SPs ranged from 9,200 to 16,000 g/mol with narrow polydispersity indices in the range of 1.4 to 1.9. As displayed in Figs. S2 to S6, the resonance peak at 2.5 parts per million (ppm) can be assigned to the proton signal of methylene linking to thiophene ring from T1-BBTD-T1 unit, while the resonance peak at ~2.8 ppm was assigned to the proton signal of methylene linking to thiophene ring from T2-BBTD-T2 unit. This result validated that T1-BBTD-T1 and T2-BBTD-T2 segments were coupled triumphantly to generate the desired SPs (SP2 to SP4). The integral ratios of the proton signals of 2.5 to ~2.8 ppm in ^1^H NMR spectra further revealed the actual composing proportion of *x*:*y* in SP2 to SP4. When the molar feed ratios of the twisted monomer to the planar monomer was 0.7:0.3, 0.5:0.5, and 0.3:0.7, respectively, the value of *x*:*y* corresponding to SP2, SP3, and SP4 was calculated to be 0.66:0.34, 0.56:0.44, and 0.34:0.66, respectively. All the obtained SPs exhibited excellent solubility in generally used organic solvents, such as toluene, dichloromethane, and tetrahydrofuran (THF), facilitating their subsequent nanoprecipitation fabrication process and applications in biological theranostics.

Next, the photophysical properties of SP1 to SP5 with various planars to twisted segmental ratios were investigated. The long-wavelength absorption bands of SP1 to SP5 in THF solutions covered the traditional NIR-I region and exhibited an evident hypochromatic shift along with the increasing ratio of the planar segment (T2-BBTD-T2) in the polymer skeleton (Fig. 3B). Because of the steric hindrance between the ortho-positioned alkyl substituent and the BBTD segment, the THF solution of SP1 showed a maximum absorption wavelength (*λ*_abs, max_) at 712 nm, while *λ*_abs, max_ of SP5 in THF solution obviously red-shifted by ~110 nm due to its higher π-conjugation extent. The *λ*_abs, max_ of SP2 to SP4 located at 746, 778, and 816 nm, respectively. In addition, the average molar extinction coefficient of SP1 to SP5 at 808 nm, an extensively used laser source in biological theranostics, was determined to be 0.69 × 10^4^, 1.09 × 10^4^_,_ 1.37 × 10^4^, 1.68 × 10^4^, and 2.03 × 10^4^ M^−1^·cm^−1^, respectively. These results were in agreement with the above theoretical calculation and further verified the positive effect of the coplanar moiety on the absorption wavelength and light-harvesting ability. More importantly, the photoluminescence (PL) spectra of these polymers covered the NIR-II window and the maximum PL peaks located at 988, 1,015, 1,021, 1,091, and 982 nm for SP1 to SP5, respectively, with their shoulder peaks extending beyond 1,250 nm (Fig. 3C). Besides, the PL properties of these SPs in aggregate states were also examined. As elucidated in Fig. 3D and Fig. S7, as the water fraction increased in THF/water mixtures, the PL intensity of SP1 and SP2 was remarkably reinforced, showing typical AIE behaviors. This phenomenon could be rationalized by their relatively distorted polymeric architectures arising from the relatively higher composition of the twisted segment T1-BBTD-T1. Nevertheless, the PL intensity of SP3 to SP5 at water fraction of 90% remained only 80%, 22%, and 24% of their PL intensity in THF solutions, respectively. The diminished aggregate-state PL performance of SP3 to SP5 may originate from the rising proportion of the largely π-conjugated coplanar T2-BBTD-T2 segment, which was favorable for the formation of harmful intrachain and/or interchain π–π interactions in dense packing manners, thus quenching the fluorescence signal. These results clearly certified the availability of the ternary copolymerization tactic via subtle regulation of the molar ratios of twisted and planar segments in precisely tuning the absorption behaviors and AIE characteristics of NIR-II SPs.

### Photophysical properties and photothermal performance of SP NPs

**Fig. 4.**
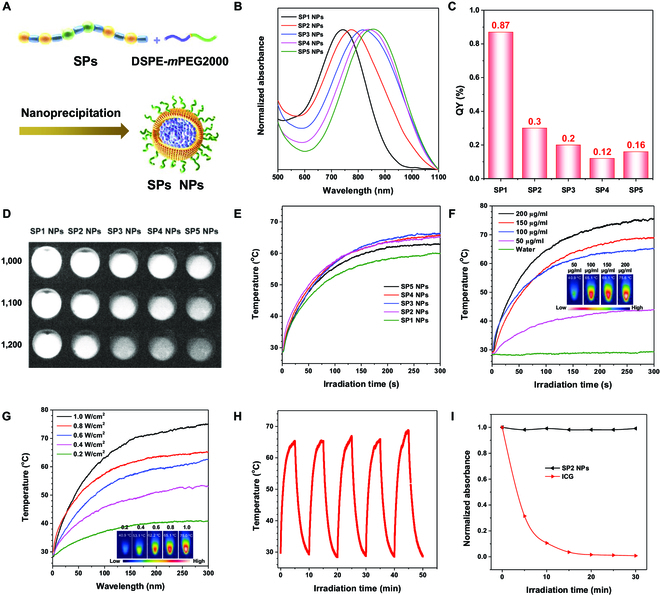
(A) Graphic interpretation of the preparation of NIR-II NPs. (B) Normalized absorption spectra of SP1 to SP5 NPs in water. (C) The QY values of SP1 to SP5 NPs (NIR-II region, 1,000 to 1,500 nm). (D) NIR-II imaging results of SP1 to SP5 NPs using 3 different LP filters. (E) Photothermal performances of SP1 to SP5 NPs (100 μg/ml) under 808-nm irradiation (0.8 W/cm^2^). (F) Photothermal performances of SP2 NPs with different concentrations under the excitation of 808-nm laser source (0.8 W/cm^2^). (G) Photothermal performances of SP2 NPs (100 μg/ml) under the excitation of 808-nm laser source with various power densities. Inset: The corresponding infrared thermal pictures. (H) Photothermal stability of SP2 NPs under 808-nm excitation. (I) Comparison of the photostability of SP2 NPs with that of ICG aqueous solution upon 808-nm irradiation.

To endow the hydrophobic polymers with excellent water dispersity and extended blood circulation competence for biological theranostics, we assembled SP1 to SP5 into NPs via the nanoprecipitation process utilizing an amphiphilic copolymer 2-distearoyl-*sn*-glycero-3-phosphoethanolamine-*N*-[methoxy(polyethylene glycol)-2000 (DSPE-*m*PEG2000) as the encapsulation matrix (Fig. 4A). Next, the photophysical properties of SP1 to SP5 were investigated. As displayed in Fig. 4B, the *λ*_abs, max_ of SP1 to SP5 NPs located at 741, 774, 818, 838, and 856 nm, respectively, possessing longer absorption wavelength compared with their corresponding THF solutions and, meanwhile, showing a gradual red-shift tendency with the increasing composition ratio of the planar T2-BBTD-T2 segment. The PL spectra of SP1 to SP5 NPs all covered the NIR-II window with an obvious tail stretching to 1,300 nm (Fig. S8), rendering their feasibility for potential NIR-II FLI in vivo. The QY values of SP1 to SP5 NPs in the NIR-II window were calculated to be 0.87%, 0.30%, 0.20%, 0.12%, and 0.16%, respectively, under 808-nm irradiation with IR-26 probe (QY = 0.5% in dichloroethane) for reference (Fig. 4C and Figs. S9 and S10). These results implied that the distorted backbone of T2-BBTD-T2 segment greatly promoted QY values. Then, the NIR-II imaging capabilities of these NPs were evaluated. As revealed in Fig. 4D, SP1 and SP2 NPs both showed strong fluorescence signal under 3 different long-pass (LP) filters, exhibiting superiority in NIR-II FLI, whereas the imaging brightness of SP3 to SP5 NPs were slightly shaded. Subsequently, the photothermal conversion abilities of the obtained NPs in aqueous solutions were estimated under 808-nm laser irradiation. Upon continuous light irradiation for 5 min at a power density of 0.8 W/cm^2^, the temperatures of SP2 to SP4 NPs all reached up to above 65 °C, while the photothermal conversion ability of SP1 NPs was slightly inferior (Fig. 4E), probably owing to its relatively weaker absorption capacity at 808 nm. As for SP5 NPs, the large π-conjugated planar T2-BBTD-T2 unit may reinforce the intrachain and/or interchain interactions of SP5 in the NPs, which could partially rigidify the structure conformation and restrain the backbone twisting motions.[57] By contrast, the marriage of planarization and twisting into one polymer may result in a looser stacking mode to benefit the intra- and interchain motions, thus favoring the heat generation within NPs. The abovementioned results suggested that the balanced modulation and ingenious integration of NIR-II FLI and photothermal properties could be achieved simply by the subtle regulation in the molar ratios of twisted and planar T-BBTD-T segments, demonstrating a potentially effective molecular engineering strategy for NIR-II FLI-assisted phototheranostics.

On the basis of the above characterization data, SP2 NPs was selected for subsequent biological applications owing to its satisfactory absorption properties, excellent NIR-II imaging quality, and high heat generation efficacy. We then conducted some additional experiments to further appraise the photothermal behaviors and stability evaluation of SP2 NPs. As displayed in Fig. 4F and G and Fig. S11, the temperature increase in SP2 NPs showed positive correlation with concentration and laser power under 808-nm excitation. The temperature of SP2 NPs at 200 μg/ml loading could reach a high plateau of 75 °C under a power density of 0.8 W/cm^2^, which was effective enough to eliminate cancer cells irreversibly in living mice [58]. The photothermal conversion efficiency (*η*) of SP2 NPs was determined to be 34% based on the “heat–cool” curve and its corresponding fitting data (Fig. S12). Noteworthily, the photothermal performances of SP2 NPs were virtually invariable after 5 cycles of “heat–cool” processes controlled by light on–off, indicating the remarkably high photothermal stability of SP2 NPs (Fig. 4H). By contrast, the heat generation performance of indocyanine green (ICG) severely attenuated after 5 laser on–off irradiation cycles (Fig. S13). Moreover, negligible absorbance variation of SP2 NPs was observed under 808-nm laser excitation for even 30 min, whereas the absorbance of ICG decreased sharply until nearly close to zero, demonstrating the excellent photobleaching resistance of SP2 NPs (Fig. 4I). Besides, the average diameter of SP2 NPs was estimated by dynamic light scattering, exhibiting around 119.3 nm with a small polydispersity index of 0.108 (Fig. S14), which is advantageous for selective tumor accumulation motivated by enhanced permeability and retention effect. In addition, the transmission electron microscopy result further disclosed the spherical morphology of the obtained SP2 NPs. Little size variation was found when SP2 NPs were contained in pure water, phosphate-buffered saline (PBS), or PBS with 10% fetal bovine serum at room temperature for 7 days (Fig. S15). These data undoubtedly testified the excellent chemical and optical stability of SP2 NPs. All of these merits rendered SP2 NPs as a promising candidate for both NIR-II FLI and PTT applications.

### In vitro tumoricidal studies

**Fig. 5.**
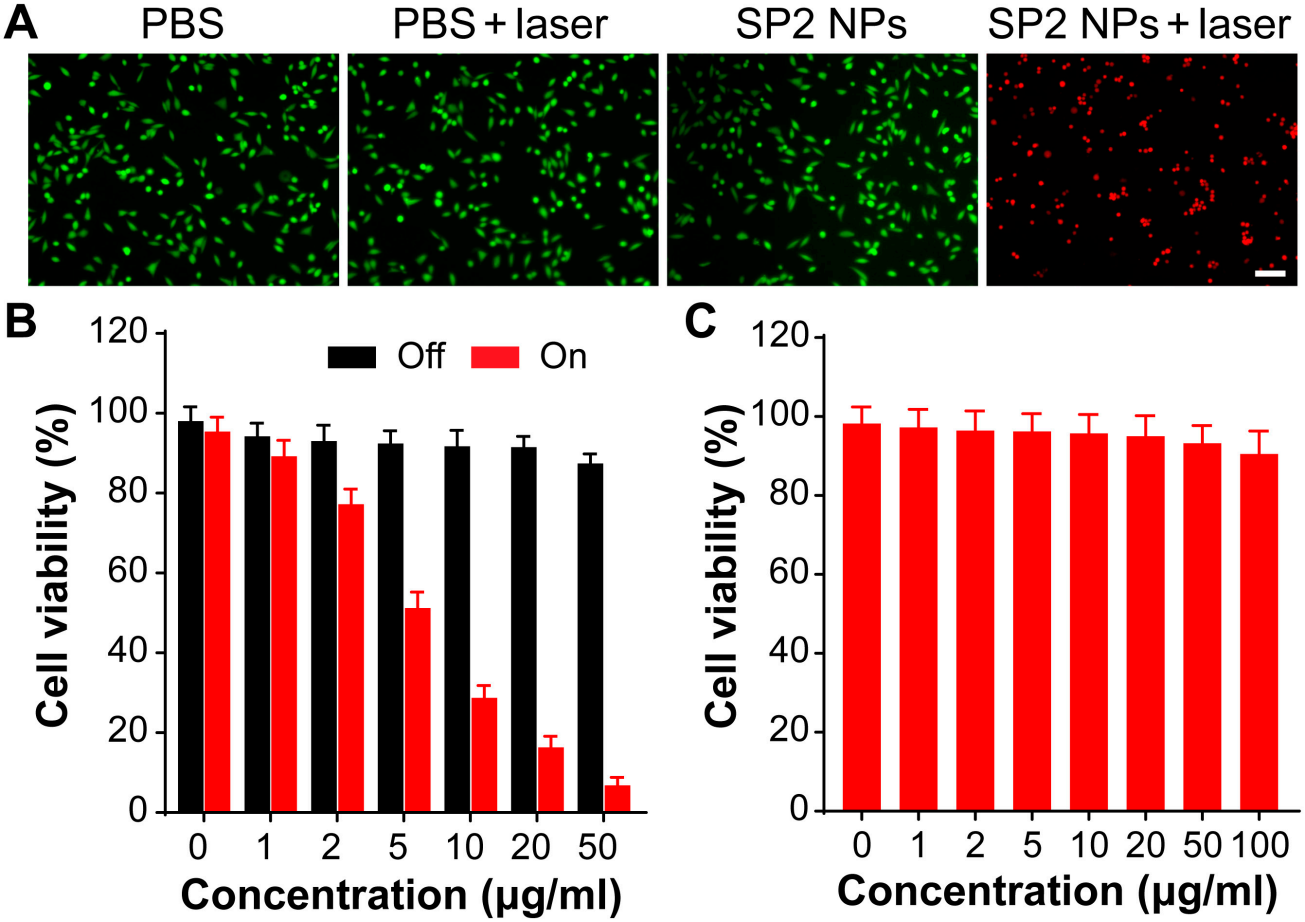
(A) Live/dead pictures of 4T1 cells after different manipulations. The green fluorescence from fluorescein diacetate denotes live cells while the red fluorescence from propidium iodide denotes dead cell. Scale bar, 50 μm. (B) Cell survival of 4T1 cells after treatment by various concentrations of SP2 NPs in the presence or absence of 808-nm laser irradiation (0.8 W/cm^2^ for 7 min, *n* = 6, means: SD). (C) Cell survival of RAW 264.7 cells after incubation with SP2 NPs at various concentrations without 808-nm excitation.

Inspired by the good photophysical properties and photothermal performance of SP2 NPs, we next investigated in vitro synergistic phototherapeutic effectiveness of SP2 NPs against mouse liver cancer 4T1 cells. Aiming to assess the in vitro PTT efficiency visually, fluorescein diacetate/propidium iodide was involved to conduct the double-staining process to differentiate live cells (green fluorescence) and dead cells (red fluorescence) by a confocal laser scanning microscopy. As displayed in Fig. 5A, strong green fluorescence was detected in PBS, PBS + laser, and SP2 NPs groups, suggesting the minimal adverse effects of the utilized laser power and the excellent biosafety of SP2 NPs. Attractively, the group using SP2 NPs joint with laser was completely stained with red fluorescence, demonstrating the excellent PTT performance and remarkable controllability of SP2 NPs toward 4T1 cells. Furthermore, the dose-dependent tumoricidal properties of SP2 NPs toward 4T1 cells were quantitatively evaluated. Without laser irradiation, SP2 NPs revealed slight influence on the cell viability even under the condition of SP2 NPs (50 μg/ml). However, upon 808-nm laser excitation for 7 min, the cell survival decreased with the increasing dose of SP2 NPs (Fig. 5B). In addition, the biocompatibility of SP2 NPs against normal RAW 264.7 cells were also evaluated using CCK-8 assay. The result in Fig. 5C showed that more than 90% RAW 264.7 cells remained alive even after incubation with SP2 NPs (100 μg/ml) under dark for 24 h, indicating the negligible cytotoxicity of SP2 NPs to normal cells. These in vitro experimental results demonstrated the great feasibility of SP2 NPs for efficient PTT tumoricidal application.

### In vivo imaging and photothermal antitumor performance

**Fig. 6.**
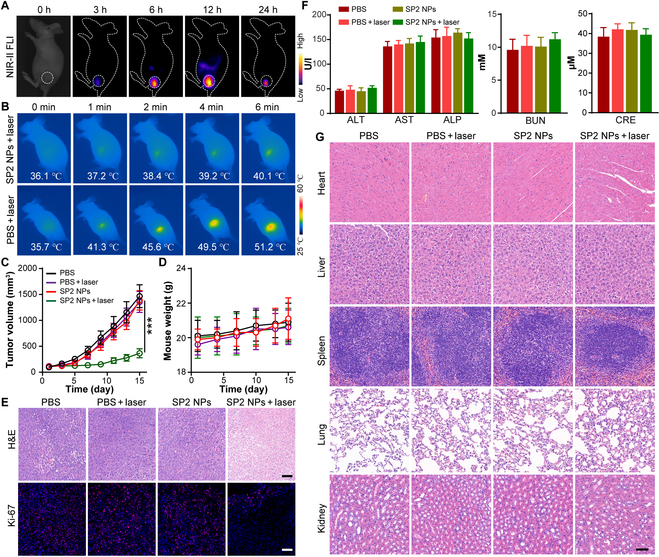
(A) NIR-II FLI results and (B) photothermal imaging results of the 4T1-tumor-bearing mice after treatment with SP2 NPs via the tail intravenous administration method at different detecting times. (C and D) Time-dependent (C) tumor growth tendency and (D) body weight tendency of the 4T1-tumor-bearing mice after treatment under different conditions. (E) H&E and Ki-67 staining results of tumor tissue of 4T1-tumor-bearing mice under various treatments. ***P < 0.001, *n* = 6. Scale bars, 100 μm. (F) Blood biochemistry indices, including hepatic function markers (left) and renal function markers (middle and right), of mice at day 30 after treatment. ALT, alanine aminotransferase; ALP, alkaline phosphatase; AST, aspartate aminotransferase; CRE, creatinine; BUN, blood urea nitrogen (BUN). (G) H&E-stained slice images of major organs after being treated under different conditions for 30 days. Scale bar, 100 μm.

The fundamental target of exploiting versatile phototheranostics is to realize precise tumor diagnosis and promoted tumoricidal effectiveness in vivo. Before the in vivo NIR-II FLI-navigated PTT evaluation of SP2 NPs, we validated their NIR-II imaging capability using different LP filters at various concentrations of SP2 NPs in aqueous solutions. The results in Fig. S16 showed obvious dose-dependent NIR-II signals, suggesting the application feasibility of SP2 NPs in NIR-II imaging in vivo. Next, the penetration depth of the NIR-II fluorescence of SP2 NPs was estimated by covering chicken breast tissues above the sample. The NIR-II fluorescence of SP2 NPs was visualized even at the chicken tissue thickness of 5 mm (Fig. S17), revealing the deep penetration superiority of NIR-II light. Subsequently, to further assess the tumorous NIR-II FLI abilities of SP2 NPs in vivo, we captured the NIR-II fluorescence images of 4T1-tumor-bearing mice after tail intravenous administration of SP2 NPs under the 808-nm laser excitation with the 1,064-nm LP filter. As revealed in Fig. 6A, the mice presented extremely faint autofluorescence before the treatment of SP2 NPs (0 h), indicating the fairly low background interference. After injecting SP2 NPs for 3 h, a rising of NIR-II fluorescence signal was captured inside the tumor region. This result suggested that the excellent accumulation of SP2 NPs in tumor section was probably motivated by the enhanced permeability and retention effect. As time prolonged, the quantified NIR-II fluorescence brightness at the tumor sections continuously enhanced and reached a maximum after 12 h of injection. Further extension of time led to a decrease in NIR-II fluorescent signal afterward due to the metabolism of NPs. In pursuit of the assessment of PTT potential of SP2 NPs, photothermal evaluations were implemented in vivo on the subcutaneous 4T1-tumor-bearing mice under the excitation of an 808-nm laser source. After the tail intravenous administration for 12 h, the tumor region was continuously irradiated by 808-nm laser for 6 min. As summarized in Fig. 6B, after treatment with SP2 NPs, the tumor region displayed a remarkable temperature elevation from 36 to 51 °C within 6 min, suggesting the high photothermal conversion efficiency of SP2 NPs inside the tumor and their great potential in photothermal tumoricidal. By contrast, merely a slight temperature elevation of 4.0 °C was observed in the PBS-injected mouse group. These results testified that SP2 NPs can efficiently accumulate at the tumor region and possessed irradiation-site-specific photothermal ablation due to the precise controllability of excitation light. In addition, the heat-caused impairment to normal organs/cells could hopefully be prevented through such intelligent phototherapeutic platform, indicating remarkable superiority over conventional chemotherapy and radiotherapy treatments.

On the basis of the remarkable NIR-II FLI ability and efficient in vivo heat generation of SP2 NPs, the PTT capability of SP2 NPs was then investigated in vivo using subcutaneous 4T1-tumor-bearing mice under the 808-nm illumination. The 4T1-tumor-bearing mice were randomly split into 4 groups, including the group of “PBS”, “PBS + laser”, “SP2 NPs”, and “SP2 NPs + laser”, respectively. As depicted in Fig. 6C, the tumor growth in the groups of “PBS”, “PBS + laser”, and “SP2 NPs” presented a steady upward tendency, implying that laser irradiation or SP2 NP injection alone exerted nearly no inhibition influence on the tumor growth. In stark contrast, the group of “SP2 NPs + laser” remarkably suppressed the tumor growth, showing outstanding PTT tumoricidal efficiency. Neither treatment-caused virtual changes in body weight nor mouse death were viewed throughout the whole treatment period (Fig. 6D), suggesting the negligible adverse effect of SP2 NPs. This result was in consistent with the excellent cytocompatibility of SP2 NPs demonstrated by the in vitro experiments. Next, the histological and immunohistochemical analysis of tumor tissues was conducted to further assess the PTT efficiency (Fig. 6E). Hematoxylin and eosin (H&E) staining of the tumor tissues from the “SP2 NPs + laser” group presented severe cell necrosis and remarkable abnormality of tumor cells, in which many hollow vacua and distinct karyopyknosis were observed. By contrast, the control groups showed little damage to the tumor tissues, and extensive and densely arranged tumor cells were observed. Similar consequence can also be drawn from the Ki-67 immunofluorescence staining analysis, in which few proliferating tumor cells were observed with the treatment of SP2 NPs and laser. All of the aforementioned results demonstrated the outstanding application prospects of SP2 NPs in the NIR-II FLI-guided PTT and in the real-time monitoring and therapeutic efficiency of tumor.

In light of the significance of therapeutic agent security in biological field, the in vivo biosafety of SP2 NPs was evaluated after tail intravenous administration of PBS or SP2 NPs into normal BALB/c nude mice under the laser irradiation treatment or not for 30 days. The blood biochemical indexes including the hepatic and renal function markers of mice injected with SP2 NPs displayed no obvious differences with the control groups (Fig. 6F), verifying the imperceptible hepatic and renal toxicity. In addition, the H&E staining results of major organs, including the heart, liver, spleen, lung, and kidney, resected from the treated mice emerged no pathological discrepancy in any of the operation groups (Fig. 6G), suggesting the low systemic toxicity of SP2 NPs. As a whole, the constructed SP2 NPs could serve as a hopeful candidate for feasible phototheranostic applications with prominent biocompatibility and biosecurity.

## Conclusions

In this assay, a precise engineering strategy of the marriage of planarization and twisting into one conjugated polymer backbone was developed, allowing for flexible tuning of the photophysical properties and photothermal conversion capacity of SPs. The twisted monomer T1-BBTD-T1 with ortho-positioned alkyl units and coplanar monomer T2-BBTD-T2 with meta-positioned alkyl units were tactfully incorporated via ternary copolymerization approach to producing SPs. By carefully regulating the feed ratio between T1-BBTD-T1 and T2-BBTD-T2 monomers, the optimal copolymer SP2 with an appropriate twisted and planar segment composition was obtained, which possessed simultaneously AIE characteristics, high absorbance at 808 nm (1.09 × 10^4^ M^−1^·cm^−1^), and satisfactory NIR-II QY (0.3%). This copolymer exhibited balanced NIR-II fluorescence output and photothermal behavior, and its corresponding water-dispersible NPs were used for theranostic applications, demonstrating prominent NIR-II FLI efficiency and photothermal ablation capability toward tumor with excellent biocompatibility as well. This work not only presents an effective SP system for NIR-II phototheranostics but also highlights an intelligent and rational molecular engineering tactic to achieve the optimal equilibrium between the NIR-II fluorescence brightness and the photothermal property in a single agent.

## Methods

Methods can be seen in the Supplementary Materials.

## Acknowledgments

**Funding:** This work was supported by the funding from the National Natural Science Foundation of China (22271197 and 22225506), the Guangdong Basic and Applied Basic Research Foundation (2023A1515011578 and 2022A1515110146), the Natural Science Foundation for Distinguished Young Scholars of Guangdong Province (2020B1515020011), Shenzhen Key Laboratory of Functional Aggregate Materials (ZDSYS20211021111400001), and the Shenzhen Science and Technology Program (RCYX20221008092924059, JCYJ20220531102601003, JCYJ20190808142403590, KQTD20210811090142053, and JCYJ20220818103007014). All animal operations in this work complied with the regulations of the Animal Ethical and Welfare Committee of Shenzhen University (AEWC-SZU). We also would like to acknowledge the Instrumental Analysis Center of Shenzhen University. **Author contributions:** X.S. contributed in the synthesis and characterization of the polymers and their NPs and wrote the manuscript draft. Z.B. conducted the biological application experiments. W.X. conducted the data analysis of biological experiments. Deliang Wang conducted the theoretical calculations. T.H. conceived the idea. T.H., Dong Wang, and B.Z.T. discussed the data, revised the manuscript, and supervised the project. All authors contributed to the article and approved the submission. **Competing interes ts:** The authors declare that they have no competing interests.

## Data Availability

All data are available in the main text or the Supplementary Materials.

## Supplementary Materials

Supplementary 1 Materials and Methods.
Table S1. Optical properties of SP1 to SP5.
Fig. S1. Synthetic routes to monomer PTZ.
Fig. S2 to S6. ^1^H NMR spectrum of SP1 to SP5, respectively.
Fig. S7. PL spectra of SP1 to SP5 in THF/water with different water fractions.
Fig. S8. Normalized PL spectra of SP1 to SP5 NPs.
Fig. S9. NIR-II QY measurement of NPs.
Fig. S10. Integrated PL spectra of the polymer samples in the region of 1,000 to 1,500 nm at various concentrations versus different absorbances at 808 nm.
Fig. S11. Thermal images of SP2 NPs with different concentrations and laser power densities.
Fig. S12. Calculation of the photothermal conversion efficiency (*η*) of SP2 NPs.
Fig. S13. Photothermal stability of ICG (100 μM) in aqueous solution.
Fig. S14. Dynamic light scattering analysis of SP2 NPs in ultrapure water.
Fig. S15. Stability analysis for size variation of SP2 NPs under different conditions.
Fig. S16. In vitro NIR-II fluorescence images of SP2 NPs with different LP filters and various concentrations.
Fig. S17. The NIR-II fluorescence signals of SP2 NPs in aqueous solutions (100 μg/ml) upon overlaying chicken tissues with different thicknesses on the top of the sample.
