# Supplementary material for: Precise Planar-Twisted Molecular Engineering to Construct Semiconducting Polymers with Balanced Absorption and Quantum Yield for Efficient Phototheranostics
Source: Research (Wash D C). 2023 Jul 26;6:0194. doi: 10.34133/research.0194 (PMC10370618; doi:10.34133/research.0194)
Supplement: Supplementary 1 — Materials and Methods. Table S1. Optical properties of SP1 to SP5. Fig. S1. Synthetic routes to monomer PTZ. Fig. S2 to S6. 1H NMR spectrum of SP1 to SP5, respectively. Fig. S7. PL spectra of SP1 to SP5 in THF/water with different water fractions. Fig. S8. Normalized PL spectra of SP1 to SP5 NPs. Fig. S9. NIR-II QY measurement of NPs. Fig. S10. Integrated PL spectra of the polymer samples in the region of 1,000 to 1,500 nm at various concentrations versus different absorbances at 808 nm. Fig. S11. Thermal images of SP2 NPs with different concentrations and laser power densities. Fig. S12. Calculation of the photothermal conversion efficiency (η) of SP2 NPs. Fig. S13. Photothermal stability of ICG (100 μM) in aqueous solution. Fig. S14. Dynamic light scattering analysis of SP2 NPs in ultrapure water. Fig. S15. Stability analysis for size variation of SP2 NPs under different conditions. Fig. S16. In vitro NIR-II fluorescence images of SP2 NPs with different LP filters and various concentrations. Fig. S17. The NIR-II fluorescence signals of SP2 NPs in aqueous solutions (100 μg/ml) upon overlaying chicken tissues with different thicknesses on the top of the sample. [file research.0194.f1.docx]

Supplementary Materials

**Precise Planar-Twisted Molecular Engineering to Construct Semiconducting Polymers with Balanced Absorption and Quantum Yield for Efficient Phototheranostics**

Xiang Su^1,2†^, Zhirong Bao^3†^, Wei Xie^1^, Deliang Wang^4^, Ting Han^1*^, Dong Wang^1*^ and Ben Zhong Tang^5*^

^1^Center for AIE Research, Shenzhen Key Laboratory of Polymer Science and Technology, Guangdong Research Center for Interfacial Engineering of Functional Materials, College of Materials Science and Engineering, Shenzhen University, Shenzhen 518060, China

^2^School of Biomedical and Pharmaceutical Sciences, Guangdong University of Technology, Guangzhou 510006, China

^3^Department of Radiation and Medical Oncology, Hubei Key Laboratory of Tumor Biological Behaviors, Hubei Cancer Clinical Study Center, Zhongnan Hospital of Wuhan University, Wuhan 430071, China

^4^Department of Materials Chemistry, Huzhou University, Huzhou 313000, China

^5^School of Science and Engineering, Shenzhen Institute of Aggregate Science and Technology, The Chinese University of Hong Kong, Shenzhen, Guangdong 518172, China

†These authors contributed equally to this work.

*Address correspondence to: hanting@szu.edu.cn; wangd@szu.edu.cn; tangbenz@cuhk.edu.cn

**1. Methods**

**Main Materials.** Unless otherwise noted, all reagents were obtained commercially and used without further purification, such as the used monomers T1-BBTD-T1 and T2-BBTD-T2 were purchased from SunaTech Inc. Phenothiazine, N-Bromosuccinimide, 1-(4-BroMophenyl)-1,2,2-triphenylethylene, *n*-BuLi, Bu_3_SnCl, Pd_2_(dba)_3_, P(*o*-tol)_3_ and tBuONa et al. were obtained from J&K, Meryer and Bide. Toluene was freshly distilled over sodium to remove H_2_O before use. All air and moisture sensitive reactions were carried out in flame-dried glassware under a nitrogen atmosphere.

**General measurements.** ^1^H NMR spectra were performed on 500 MHz NMR spectrometers (Bruker AVANCE). Chemical shifts were calibrated using CDCl_3_ (^1^H NMR: δ 7.26 ppm) as internal reference. *M*_w_, *M*_n_ and polydispersity indices (PDI, *M*_w_/*M*_n_) of the obtained polymers were estimated by Waters 1525 gel permeation chromatography system with THF as eluent using reactive index (RI) detector and calibrated using a set of monodispersed polystyrene standards. UV-Vis-NIR absorption spectra were measured on a PerkinElmer Lambda 950 spectrophotometer. PL spectra were recorded on FLS1000 Photoluminescence Spectrometer of Edinburgh Instruments. Particle size analyses were implemented using a Zetasizer Nano ZSP (Malvern NanoZSP + MPT-2). Photothermal experiments were implemented by using an 808 nm infrared semiconductor laser (Changchun radium photoelectric technology). Temperature changes were recorded by an E6 IR thermal camera (FLIR Systems). CCK-8 assays were conducted on a BioTek microplate reader. Density functional theory (DFT) calculations were carried out by the B3LYP/6G(d), Gaussian 09 package.

**Fabrication of semiconducting polymer (SP) nanoparticles (NPs).** A clear THF (1 mL) solution of conjugated polymer (1 mg), DSPE-mPEG2000 (5.0 mg) was quickly injected into 9 mL of deionized water, which was sonicated with a microtip probe sonicator (XL2000, Misonix Incorporated, NY) at 45% output power for 2 min continuously. Then the mixtures were transferred into dialysis tube (MWCO 3500 Da) and dialyzed against deionized water for 24 h. In order to remove THF completely, the water was replaced by fresh water every 4 h. The final obtained nanoparticle solutions were concentrated by ultrafiltration before use. The corresponding concentration of each SP NPs was calculated using a pre-established calibration absorption curve.

**Determination of quantum yield (QY) of SP NPs.** The QY of the dyes was measured in a similar way to the previous report ^[1]^, using NIR-II fluorescent IR-26 dye as the reference (QY = 0.5%). For reference calibration, IR-26 dissolved in 1,2-dichloroethane (DCE) was diluted to a DCE solution to prepare five samples with their absorbance value at 808 nm of ~0.1, ~0.08, ~0.06, ~0.04, and ~0.02. The PL spectra were collected from 850 nm to reject the excitation light (808 nm). Then the emission spectra were integrated in the 1000‒1500 nm NIR-II region. The same procedures were performed for the nanoparticles of SP1‒SP5 in water. The integrated NIR-II fluorescence intensity was plotted against absorbance at the excitation wavelength of 808 nm and fitted into a linear function. The QY calculation equation was as follows:

where QY_sample_ is the QY of the nanoparticles in 1000-1500 nm, QY_ref_ is the QY of IR-26 (0.5% in dichloroethane), S_sample_ and S_ref_ refer to the slopes obtained by linear fitting of the integrated emission spectra of the nanoparticles (1000‒1500 nm) and IR-26 (1000‒1500 nm) against the absorbance at 808 nm, *n*_sample_ and *n*_ref_ are the refractive indices of water and DCE, respectively.

**Photothermal performance measurement of SP NPs.** The aqueous solutions of nanoparticles SP1 NPs-SP5 NPs with the specified concentrations were continuously exposed to an 808 nm laser at appointed power density. The temperature was measured every 5s and stopped until the temperature nearly reached to a plateau. The corresponding infrared thermal images of the sample tubes were also acquired. Pure water under the same condition served as the control groups. In addition, the photothermal conversion efficiency (η) of SP2 NPs in aqueous solution was calculated according to the literature. ^[2-3]^

**In vitro cytotoxicity study.** CCK-8 assays were employed to evaluate the photothermal killing ability of SP2 NPs against 4T1 cells. In brief, 4T1 cells were seeded in 96-well plates at a density of 5 × 10^3^ cells per well. After 24 h incubation, the medium was replaced by fresh medium containing a series of doses (0, 1, 2, 5, 10, 20 and 50 μg/mL based on SP2) of SP2 NPs. After incubated for 12 h, the cells of light groups were treated with 808 nm laser irradiation (0.8 W/cm^2^, 7 min), and the dark groups were kept in dark. After another 24 incubations, the old medium was removed and 100 μL of fresh medium containing 10% CCK without serum were added into each well. After 1 h incubation in the dark, the absorbance of CCK-8 at 450 nm was monitored by the microplate Reader. The viability of cells was expressed as the percentage of cells after different treatments relative to the control cells without any treatment.

**Cell cytotoxicity evaluation.** RAW 264.7 cells were seeded in a 96-well plate with a density of 5 × 10^5^ cells per well. After 24 h incubation, different concentrations of SP2 NPs were added in the wells and incubated with cells for another 24 h. CCK-8 assay was used to stain the cells and the cytotoxicity was measured by microplate reader (excitation wavelength = 570 nm).

**Live-dead cell staining.** The 4T1 cells were seeded and cultured in glass bottom dish for 24 h, SP2 NPs (50 μg/mL) was then added into the cell culture medium. After 12 h incubation, the cells were washed and replaced with fresh medium, followed by 808 nm laser irradiation (0.8 W/cm^2^) for 7 min. After that, the cells were incubated at 37 °C for another 1 h, then successively stained with PI (60 μg/mL) and FDA (100 μg/mL) in PBS for 10 min. Subsequently, the cells were gently washed and then imaged by CLSM. Conditions: excitation wavelength: 488 nm for FDA and 534 nm for PI; emission filter: 500-550 nm for FDA and 550-650 nm for PI.

**Animals and tumor models.** The 3 weeks of BALB/c nude mice were purchased from Beijing Vital River Laboratory Animal Technology. All animals were acclimatized to the animal facility for one week prior to experimentation and housed under pathogen free conditions. All animals were fed under conditions of 25 °C and 55% of humidity and allowed free access to standard laboratory water and chow. All the animal procedures were performed according to the guidelines of the Animal Ethical and Welfare Committee of Shenzhen University. The xenograft tumor models were established by subcutaneous injection of 1 × 10^6^ 4T1 into the right flanks of each mouse.

**In vivo NIR-II fluorescence and photothermal imaging.** The 4T1 tumor-bearing mice were administered with SP2 NPs in saline at a dose of 200 μg SP2 per mouse via tail vein. Then, at 3, 6, 12 and 24 h post-injection, the mice were anesthetized using 2% isoflurane in oxygen and underwent NIR-II fluorescence imaging through a commercial measurement purchased from Suzhou NIR-Optics Technologies CO., Ltd., with the long pass (LP) filter of 1000 nm. For in vivo photothermal imaging, the infrared thermal images of mice were acquired using an E6 IR camera during the irradiation of 808 nm laser (0.8 W/cm^2^) for 6 min at 12 h after administration with SP2 NPs. Mouse injected with saline under the same irradiation condition was used as the control.

**In vivo phototherapeutic study.** When the inoculated tumor grew for 8 d, 24 mice were randomly divided into 4 groups, named “PBS”, “SP2 NPs” “PBS + L”, and “SP2 NPs + L”, respectively. On day 0, for “PBS” and “SP2 NPs” groups, 200 μL of PBS and SP2 NPs (200 μg SP2) were separately injected into the 4T1 tumor-bearing mice through tail vein without subsequent laser irradiation. In case of “PBS + L” and “AIE NPs + L” groups, after intravenous injection 200 μL of PBS and SP2 NPs (200 μg SP2) for 12 h, respectively, the tumors of mice in each group were continuously irradiated with 808 nm laser (0.8 W/cm^2^) for 7 min. After a variety of treatments, the mouse body weight and tumor volume were recorded every 3 days during 15-day study duration. The tumor volume was measured by a vernier caliper and calculated as V = a × b^2^/2 (a: tumor length; b: tumor width). In addition, the tumor tissues were then subjected to H&E staining and immunohistochemical studies.

**In vivo toxicity evaluation.** Blood samples were harvested from the healthy mice to measure the levels of ALT (alanine aminotransferase), AST (aspartate aminotransferase), ALP (alkaline phosphatase), CRE (creatinine) and BUN (blood urea nitrogen). Major organs (hearts, livers, spleens, lungs, and kidneys) were also collected and examined by H&E staining.

**Statistical analysis.** All numeric data are expressed as mean ± s.d. unless otherwise indicated. For multiple comparisons, one-way analysis of variance (ANOVA) with Tukey’s post hoc test was used. Statistical analysis was performed using GraphPad Prism 6.0. P values of less than 0.05 were considered significant. *P < 0.05, **P < 0.01, ***P < 0.001 and ****P < 0.0001.

**2. Synthetic procedures and characterization data for the SPs**

Monomer PTZ was synthesized according to the previous reported method ^[4-6]^ and the synthetic procedure can be briefly described as Scheme S1.

**Figure S1.** Synthetic routes to monomer PTZ.

Five conjugated polymers SP1-SP5 were synthesized with the same procedures by varying the feed molar ratios of monomers PTZ, T1-BBTD-T1 and T2-BBTD-T2 as below.^[7]^

**Synthesis of SP1**. Under N_2_ atmosphere, PTZ (111 mg, 0.1 mmol), T1-BBTD-T1 (74 mg, 0.1 mmol), Pd_2_(dba)_3_ (9.2 mg, 0.01 mmol), P(*o*-tol)_3_ (24.3 mg, 0.08 mmol) and 1 mL toluene were added to a 10 mL pre-dried tube. The mixture was refluxed at 120 ^o^C for 24 h. After cooling down to room temperature, the resulting solution was diluted with 1 mL DCM and precipitated into methanol (200 mL) through a neutral Al_2_O_3_-filled dropper to remove the catalyst. The precipitate was finally collected after filtration, being washed with methanol, and dried under vacuum at 50 ^o^C to obtain the target product SP1 (yield, 78%) (*M*_w_ = 15500 g/mol, PDI = 1.9).

**Synthesis of SP2**. Under N_2_ atmosphere, PTZ (111 mg, 0.1 mmol), T1-BBTD-T1 (52 mg, 0.07 mmol), T2-BBTD-T2 (32 mg, 0.03 mmol), Pd_2_(dba)_3_ (9.2 mg, 0.01 mmol), P(*o*-tol)_3_ (24.3 mg, 0.08 mmol) and 1 mL toluene were added to a 10 mL pre-dried tube. The mixture was refluxed for 24 h. After cooling down to room temperature, the resulting solution was diluted with 1 mL DCM and precipitated into methanol (200 mL) through a neutral Al_2_O_3_-filled dropper to remove the catalyst. The precipitate was finally collected after filtration, being washed with methanol, and dried under vacuum at 50 ^o^C to obtain the target product SP2 (yield, 70%) (*M*_w_ = 13700 g/mol, PDI = 1.8).

**Synthesis of SP3**. Under N_2_ atmosphere, PTZ (111 mg, 0.1 mmol), T1-BBTD-T1 (37 mg, 0.05 mmol), T2-BBTD-T2 (54 mg, 0.05 mmol), Pd_2_(dba)_3_ (9.2 mg, 0.01 mmol), P(*o*-tol)_3_ (24.3 mg, 0.08 mmol) and 1 mL toluene were added to a 10 mL pre-dried tube. The mixture was refluxed for 24 h. After cooling down to room temperature, the resulting solution was diluted with 1 mL DCM and precipitated into methanol (200 mL) through a neutral Al_2_O_3_-filled dropper to remove the catalyst. The precipitate was finally collected after filtration, being washed with methanol, and dried under vacuum at 50 ^o^C to obtain the target product SP3 (yield, 67%) (*M*_w_ = 11000 g/mol, PDI = 1.6).

**Synthesis of SP4**. Under N_2_ atmosphere, PTZ (111 mg, 0.1 mmol), T1-BBTD-T1 (22 mg, 0.03 mmol), T2-BBTD-T2 (75 mg, 0.07 mmol), Pd_2_(dba)_3_ (9.2 mg, 0.01 mmol), P(*o*-tol)_3_ (24.3 mg, 0.08 mmol) and 1 mL toluene were added to a 10 mL pre-dried tube. The mixture was refluxed for 24 h. After cooling down to room temperature, the resulting solution was diluted with 1 mL DCM and precipitated into methanol (200 mL) through a neutral Al_2_O_3_-filled dropper to remove the catalyst. The precipitate was finally collected after filtration, being washed with methanol, and dried under vacuum at 50 ^o^C to obtain the target product SP4 (yield, 63%) (*M*_w_ = 9200 g/mol, PDI = 1.5).

**Synthesis of SP5**. Under N_2_ atmosphere, PTZ (111 mg, 0.1 mmol), T2-BBTD-T2 (107 mg, 0.1 mmol), Pd_2_(dba)_3_ (9.2 mg, 0.01 mmol), P(*o*-tol)_3_ (24.3 mg, 0.08 mmol) and 1 mL toluene were added to a 10 mL pre-dried tube. The mixture was refluxed for 24 h. After cooling down to room temperature, the resulting solution was diluted with 1 mL DCM and precipitated into methanol (200 mL) through a neutral Al_2_O_3_-filled dropper to remove the catalyst. The precipitate was finally collected after filtration, being washed with methanol, and dried under vacuum at 50 ^o^C to obtain the target product SP5 (yield, 60%) (*M*_w_ = 5500 g/mol, PDI = 1.4).

The structures of SP1-SP5 were identified by the ^1^H NMR technique. As presented in Figure S1-S5, the integral ratios of the proton signals of 2.5 ppm to ~2.8 ppm could reveal the composing proportion of x:y in copolymers SP2‒SP4.

**Figure S2.** The ^1^H NMR spectrum of SP1.

**Figure S3.** The ^1^H NMR spectrum of SP2.

**Figure S4.** The ^1^H NMR spectrum of SP3.

**Figure S5.** The ^1^H NMR spectrum of SP4.

**Figure S6.** The ^1^H NMR spectrum of SP5.

**Table S1**. Optical properties of SP1‒SP5.

| Sample | Solution | | | Nanoparticle | *α*_AIE_ *^f^* |
| --- | --- | --- | --- | --- | --- |
|  | *λ*_abs_[nm]*^a^* | *ε* [×10^4^ M^-1^ cm^-1^]*^b^* | *λ*_em_ [nm]*^c^* | *λ*_abs_ [nm]*^d^* (QY)*^e^* |  |
| SP1 | 712 | 0.69 | 988 | 741 (0.87%) | 2.42 |
| SP2 | 746 | 1.09 | 1015 | 774 (0.30%) | 3.02 |
| SP3 | 778 | 1.37 | 1021 | 818 (0.20%) | 0.80 |
| SP4 | 816 | 1.68 | 1091 | 838 (0.12%) | 0.22 |
| SP5 | 818 | 2.03 | 982 | 856 (0.16%) | 0.25 |

*^a^* The maximum absorption wavelength of SP1‒SP5 in their THF solutions. *^b^* The average molar extinction coefficient of the polymer samples in their THF solutions (10 μM) at 808 nm. *^c^* The maximum emission wavelength of the polymer samples in their THF solutions. *^d^* The maximum absorption wavelength of SP1‒SP5 NPs in aqueous solutions. *^e^* Relative fluorescence quantum yields of SP1‒SP5 NPs in aqueous solutions using IR-26 as reference. *^e^α*_AIE_ = *I*_90_/*I*_0_.


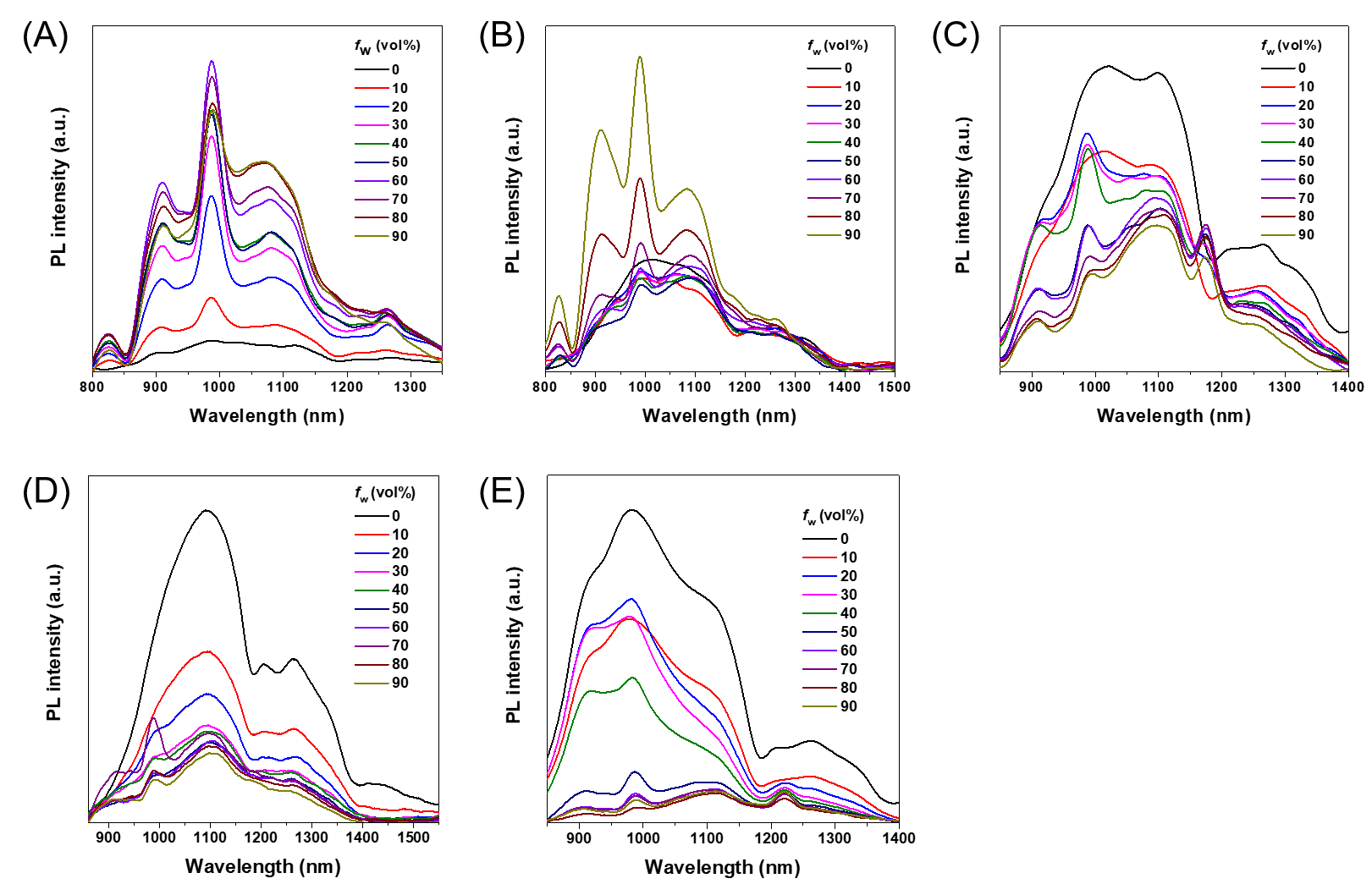


**Figure S7.** PL spectra of (A) SP1, (B) SP2, (C) SP3, (D) SP4, and (E) SP5 in THF/water with different water fractions (*f*_W_); Concentration: 10 μΜ.

**Figure S8.** Normalized PL spectra of SP1‒SP5 NPs.


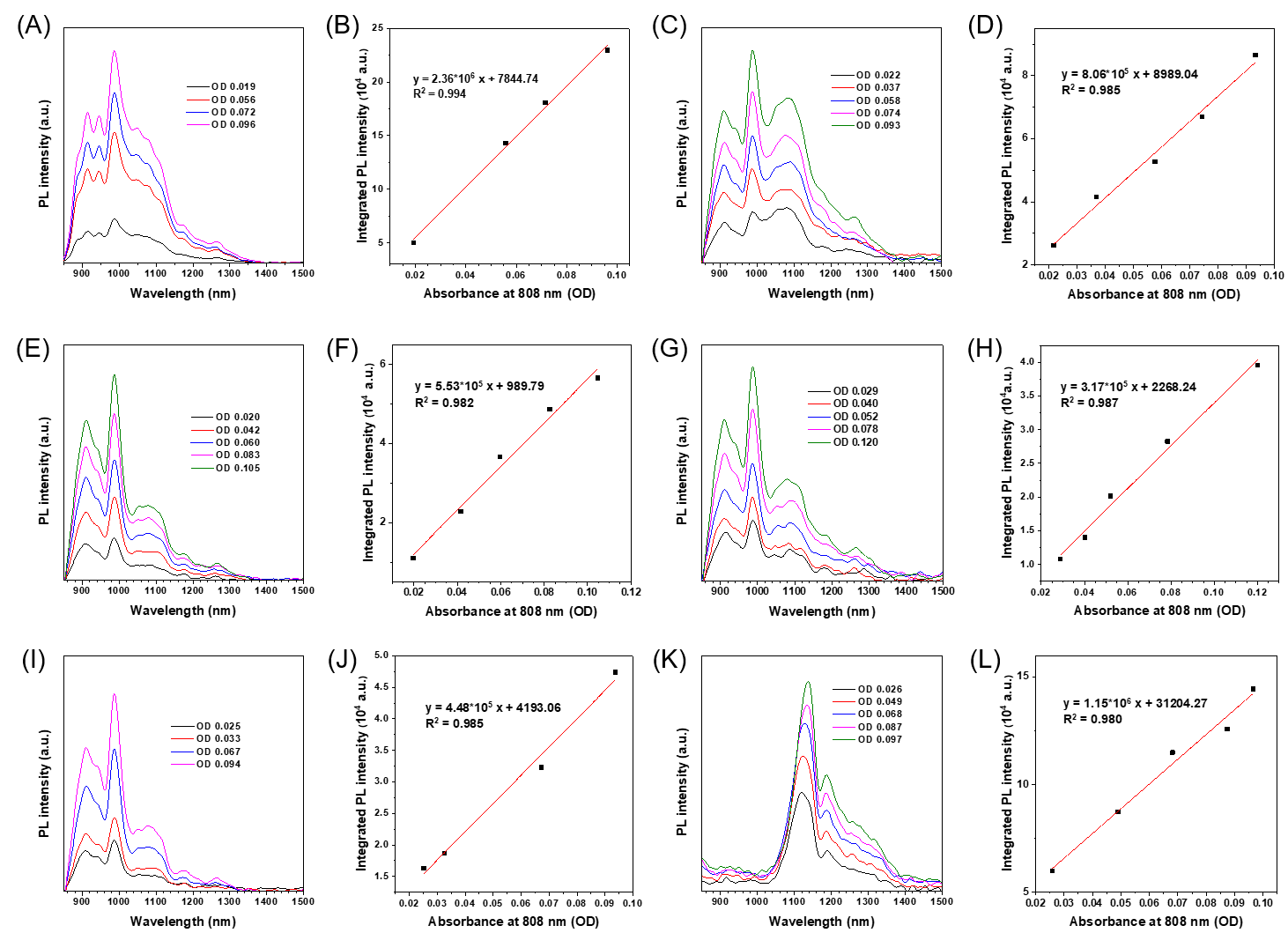


**Figure S9.** NIR-II quantum yield measurement of nanoparticles. PL spectra and a plot of integrated fluorescence intensity NIR-II (1000-1500 nm) *vs* the absorbance at 808 nm of (A, B) SP1 NPs in ultrapure water, (C, D) SP2 NPs in ultrapure water, (E, F) SP3 NPs in ultrapure water, (G, H) SP4 NPs in ultrapure water, (I, J) SP5 NPs in ultrapure water and (K, L) IR-26 in DCE solution.


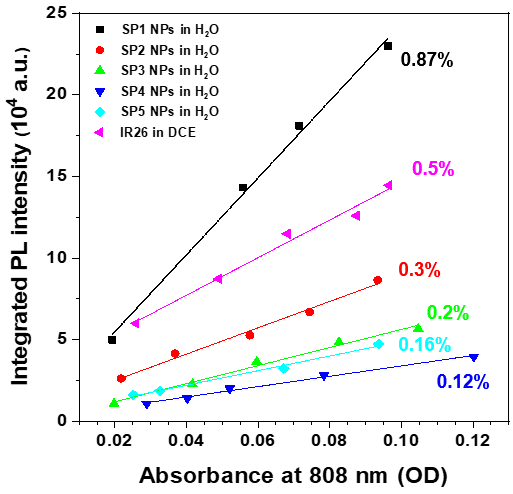


**Figure S10.** Integrated PL spectra of the polymer samples in the region of 1000‒1500 nm at various concentrations versus different absorbance at 808 nm (IR-26, QY = 0.5% in DCE).


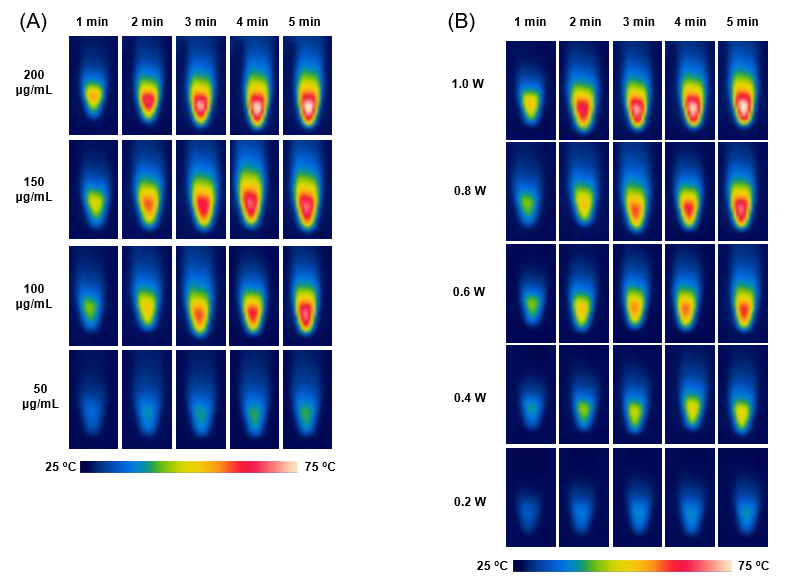


**Figure S11.** (A) Thermal images of SP2 NPs at various concentrations under 808 nm laser irradiation (0.8 W/cm^2^). (B) Thermal images of SP2 NPs (100 μg/mL) upon 808 nm laser irradiation with different power densities.


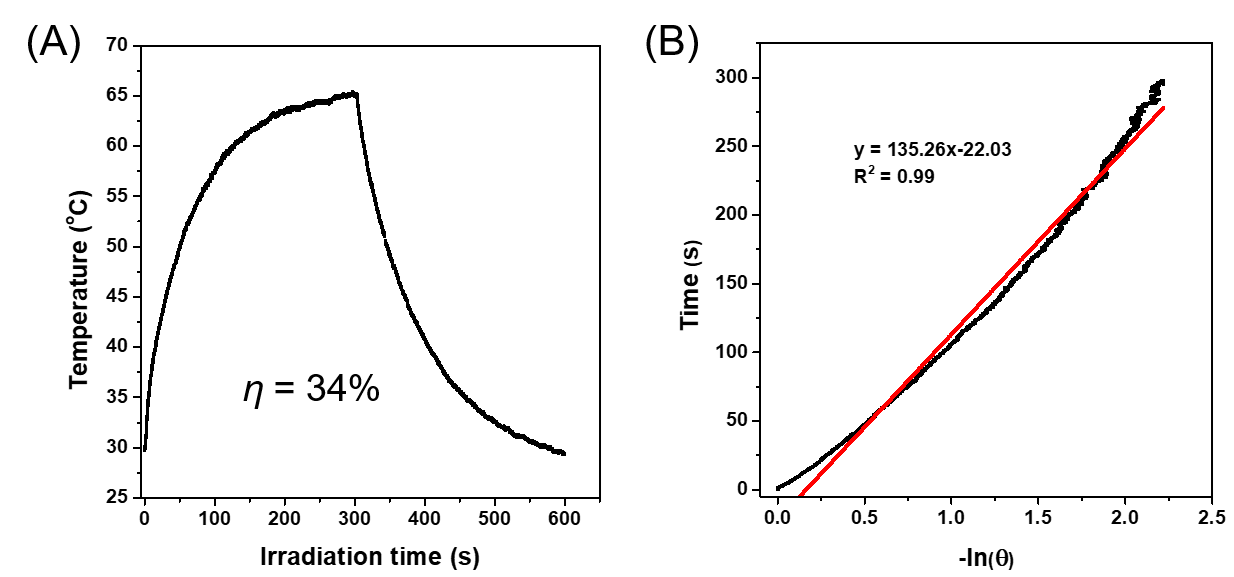


**Figure S12.** Temperature profile of SP2 NPs irradiated by 808 nm laser (0.8 W/cm^2^) for 5 min, followed by natural cooling. (B) The linear fitting of time from the cooling period versus negative natural logarithm of driving force temperature for SP2 NPs.


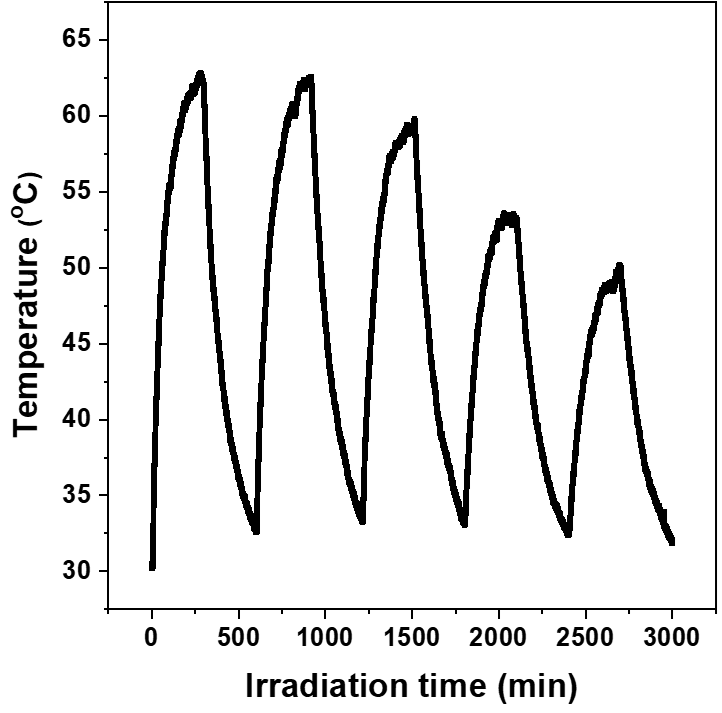


**Figure S13.** Photothermal stability of indocyanine green (ICG, 100 μM) in aqueous solution during five on/off irradiation cycles with an 808 nm laser at a power density of 0.8 W/cm^2^.

**Figure S14.** DLS analysis and represent TEM image (inset) of SP2 NPs in ultrapure water. Scar bar: 100 nm.


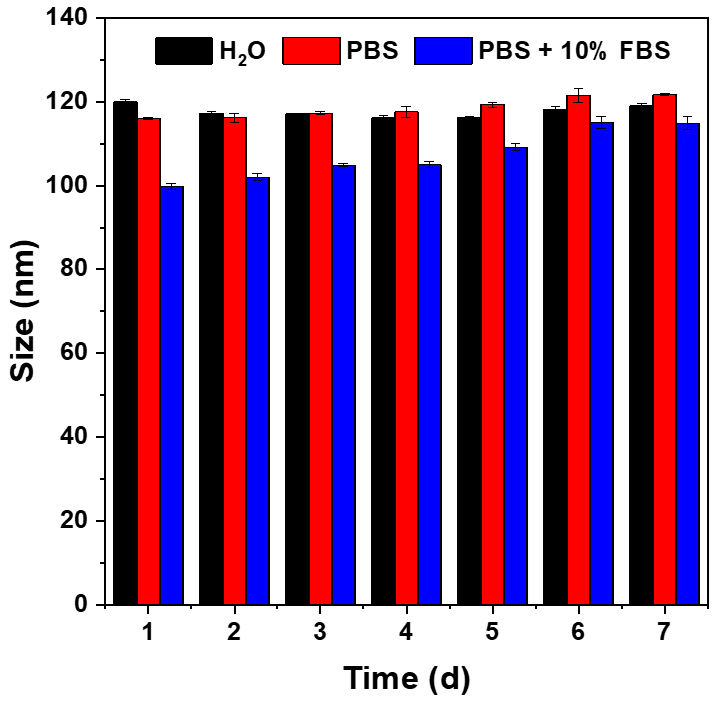


**Figure S15.** Stability analysis for the size variation of SP2 NPs with a concentration of 100 μg/mL at room temperature in ultrapure water, PBS or PBS + 10% FBS measured by DLS (means: SD, n = 3).

**Figure S16.** In vitro NIR-II fluorescence images of SP2 NPs with different LP filters and various concentrations.


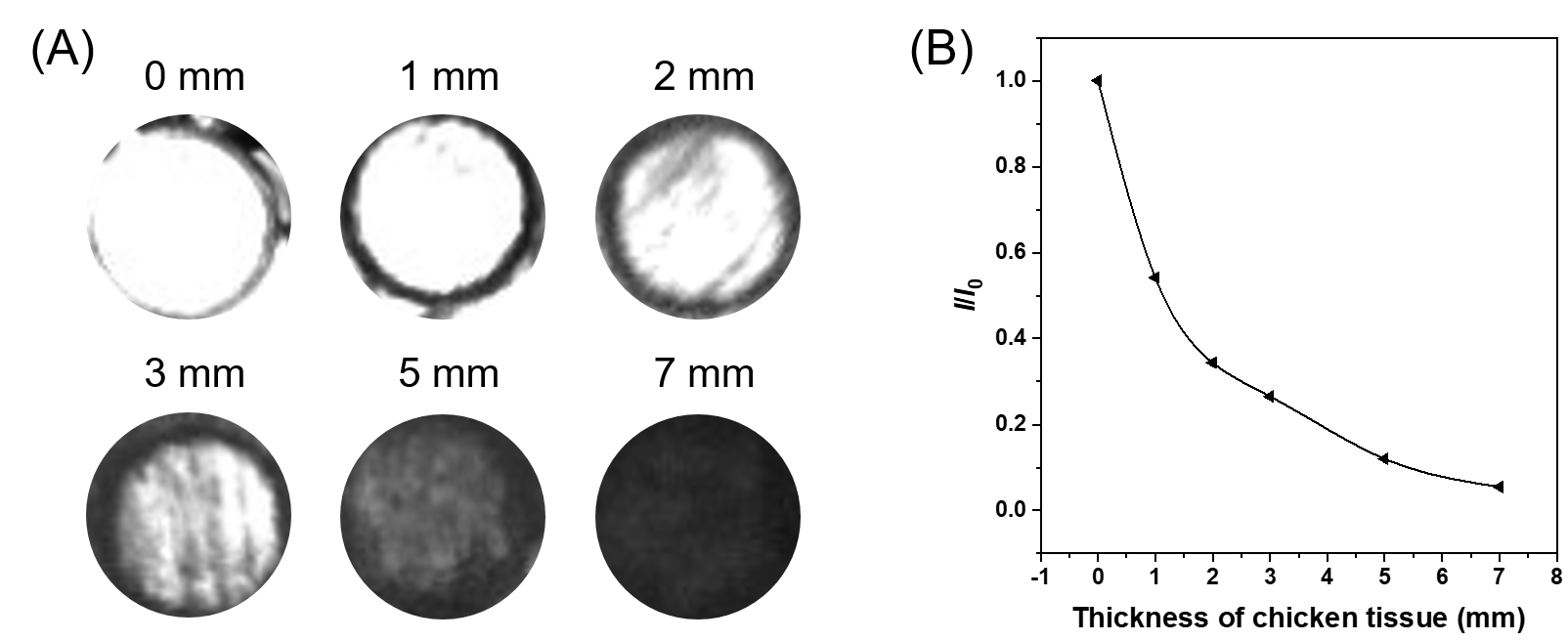


**Figure S17.** (A) Representative NIR-II fluorescence images of SP2 NPs in aqueous solutions (100 µg/mL) upon overlaying chicken tissues with different thickness on top of the sample upon excitation at 808 nm. (B) Relative PL intensity of SP2 NPs with the increasing thickness of chicken tissue.

**Supplementary references**

[1] D. Yan, W. Xie, J. Zhang, L. Wang, D. Wang, B. Z. Tang, *Angew. Chem. Int. Ed*. **2021**, *60*, 26769-26776.

[2] D. K. Roper, W. Ahn, M. J. Hoepfner, *Phys. Chem. C*, **2007**, *111*, 3636-3641.

[3] Q. Tian, F. Jiang, R. Zou, Q. Liu, Z. Chen, M. Zhu, S. Yang, J. Wang, J. Wang, J. Hu, *ACS Nano* **2011**, *5*, 9761-9771.

[4] A. C. B. Rodrigues, J. Pina, W. Dong, M. Forster, U. Scherf, J. Sergio Seixas de Melo, *Macromolecules* **2018**, *51*, 8501−8512.

[5] Z. Zhang, X. Fang, Z. Liu, H. Liu, D. Chen, S. He, J. Zheng, B. Yang, W. Qin, X. Zhang, C. Wu, *Angew. Chem. Int. Ed*. **2020**, *59*, 3691-3698.

[6] Z. Zhang, D. Chen, Z. Liu, D. Wang, J. Guo, J. Zheng, W. Qin, C. Wu, *ACS Appl. Polym. Mater.* **2020**, *2*, 74−79.

[7] S. Liu, H. Ou, Y. Li, H. Zhang, J. Liu, X. Lu, R. T. K. Kwok, J. W. Y. Lam, D. Ding, B. Z. Tang, *J. Am. Chem. Soc.* **2020**, *142*, 15146–15156.
